# Full-length transcriptome analysis provides new insights into the early bolting occurrence in medicinal *Angelica sinensis*

**DOI:** 10.1038/s41598-021-92494-4

**Published:** 2021-06-21

**Authors:** Xue Gao, Fengxia Guo, Yuan Chen, Gang Bai, Yuxiao Liu, Jianqin Jin, Qing Wang

**Affiliations:** 1 College of Life Science and Technology, Gansu Provincial Key Lab of Arid Land Crop Science, Gansu Key Lab of Crop Genetic and Germplasm Enhancement, Gansu Agricultural University, Lanzhou, 730070 China; 2 College of Agronomy, Gansu Provincial Key Lab of Good Agricultural Production for Traditional Chinese Medicines, Gansu Provincial Engineering Research Centre for Medical Plant Cultivation and Breeding, Gansu Agricultural University, Lanzhou, 730070 China

**Keywords:** Transcription, Transcriptomics, Bioinformatics, Gene expression analysis, Sequencing, Functional genomics, Gene regulation

## Abstract

*Angelica sinensis* (Oliv.) Diels root part is an integral component of traditional Chinese medicine, widely prescribed to improve blood circulation and blood stasis. However, early bolting of *A. sinensis* compromises the quality of the roots and hence is a major limitation for yield of medicinal materials. To date, little information about the molecular mechanisms underlying bolting is available for this important medicinal plant. To identify genes putatively involved in early bolting, we have conducted the transcriptome analysis of the shoot tips of the early-bolting plants and non-bolting (normal) plants of *A. sinensis*, respectively, using a combination of third-generation sequencing and next-generation sequencing. A total of 43,438 non-redundant transcripts were collected and 475 unique differentially expressed genes (DEGs) were identified. Gene annotation and functional analyses revealed that DEGs were highly involved in plant hormone signaling and biosynthesis pathways, three main flowering pathways, pollen formation, and very-long-chain fatty acids biosynthesis pathways. The levels of endogenous hormones were also changed significantly in the early bolting stage of *A. sinensis*. This study provided new insights into the transcriptomic control of early bolting in *A. sinensis,* which could be further applied to enhance the yield of medicinally important raw materials.

## Introduction

*Angelica sinensis* (Oliv.) Diels (Apiaceae), known as *Danggui* in China, is a traditional Chinese medicinal herb^1,2^. Its dried roots have usually been used in traditional Chinese medicine to replenish and invigorate blood, lubricate the intestines, and treat irregular menstruation^3,4^. Modern pharmacological studies have demonstrated that *A. sinensis* exhibits anti-tumor and anti-arrhythmic activities, enhancing the immune system, and scavenges free radicals through antioxidant activity^5–8^. Wild *A. sinensis* naturally grow in alpine mountainous habitats at high elevations, and due to its scarcity, the majority of the herb supply now comes from the cultivated stock^9^. Gansu province is the largest supplier of *A. sinensis* in China, accounting for approximately 90% of the herb in the domestic market^10^. As the demand for *A. sinensis* increases in international markets, its production has become a critical pillar to the local economy in Gansu, China.

However, the continued development of the *A. sinensis* industry is hindered by early bolting^11^, a phenomenon where the plant prematurely transitions from vegetative growth to reproductive growth phase, ultimately leading to early flowering and seed set. Flowering severely affects the accumulation of medicinal compounds in *A. sinensis* roots as nutrients are diverted to the floral shoot; consequently, the accumulation of secondary metabolites in roots is reduced, leading to a decrease in the medicinal and nutritional value of the root^12^. Limiting the prevalence of early bolting is therefore critical to improving the production of high-quality *A. sinensis* roots. Previous investigations towards the understanding and reduction of early bolting in *A. sinensis* mainly focused on physiology and ecology aspects^13,14^. Few reports provide evidence on the molecular mechanisms underlying early bolting in *A. sinensis,* and the related genes are largely unknown.

In recent years, third-generation sequencing (TGS) technologies, such as the Single-Molecule Real-Time (SMRT) sequencing platform from Pacific Biosciences (Pacific Biosciences, CA, USA) enable the rapid identification of genes and molecular mechanisms underlying crucial crop phenology. These technologies facilitate gene discovery in non-model species, such as traditional medicinal crops for which published reference genomes may not exist^15–17^. Furthermore, the improved read lengths of TGS platforms render it advantageous to next-generation sequencing (NGS), greatly reducing the difficulty of transcriptome analysis^18^.

To explore the full-length transcriptome of *A. sinensis* and transcriptomic differences between an early bolting genotype and a normal bolting genotype during bolting, the present study is performed using a combination of SMRT and NGS sequencing technologies. Differentially expressed genes (DEGs) were identified and key genes which involved in early bolting were assessed. Moreover, changes in endogenous hormone levels were also detected during bolting. The obtained transcriptome data enable further exploration into the molecular mechanism of early bolting and growth regulation of important medicinal plants in the Apiaceae family.

## Results

### Full-length transcriptome sequencing

**Figure 1:**
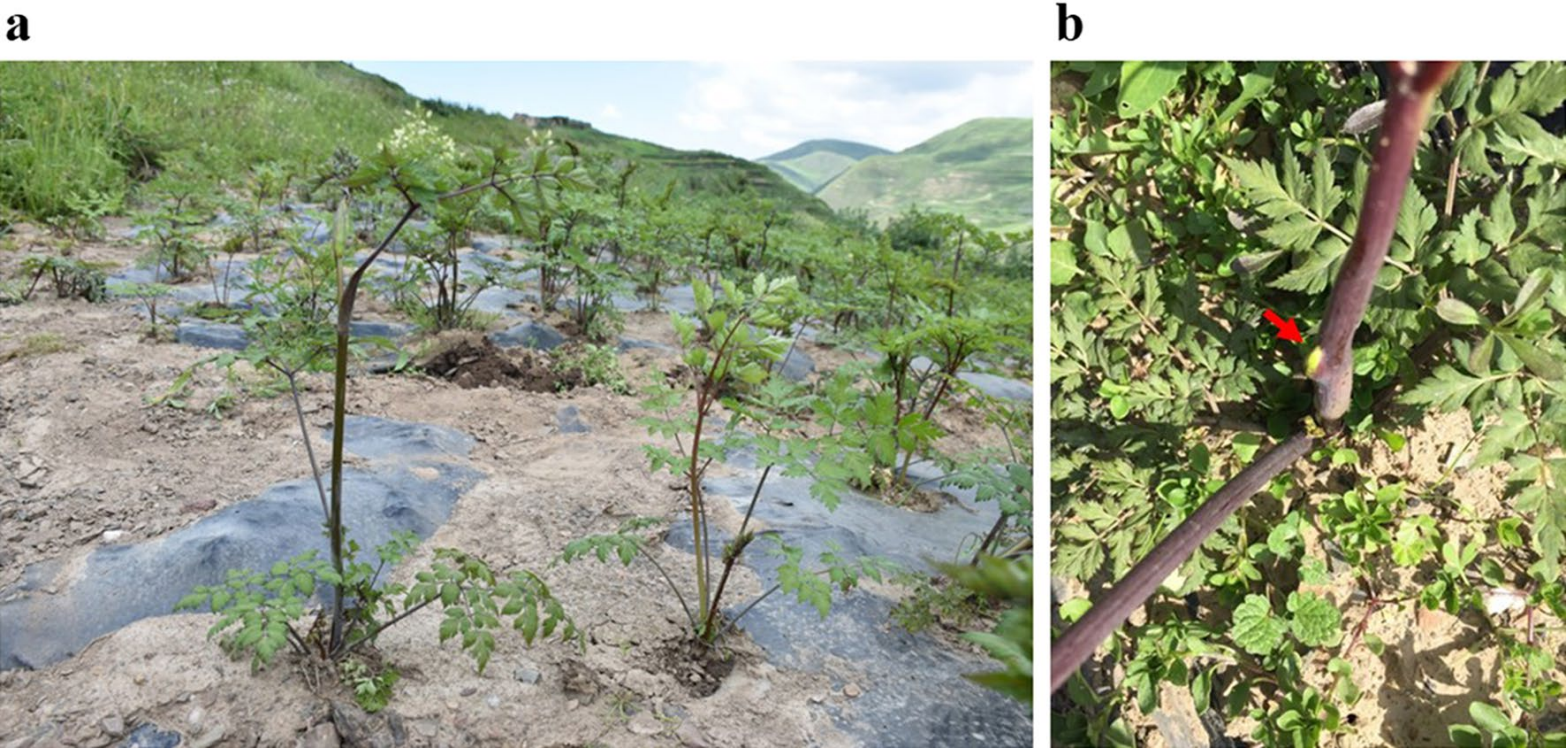
*A. sinensis* cultivated in Min County, Gansu Province, China. (**a**) Bolting plants (BP) and normal plants (NP) in the same growth period; The whole growth period of *A. sinensis* typically consists of 3 years, including of 2 years vegetative growth, followed by flowering and seed set from the third year onwards. In early bolting ecotypes, flowering occurs prematurely in the second year. (**b**) Sampling position. Shoot tips of *A. sinensis* under the same growth conditions were collected before bolting.

**Figure 2:**
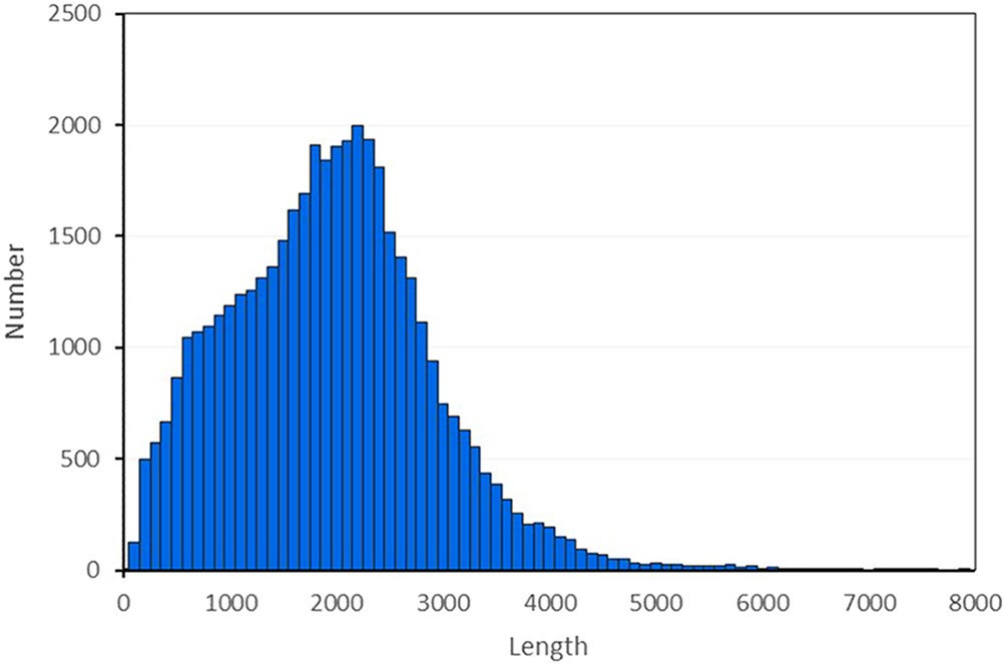
Length distribution of *A. sinensis* transcripts generated by single-molecule long-read sequencing.

Two sequencing technologies, Illumina NGS sequencing and PacBio SMRT sequencing were employed for full-length transcriptome analysis of *A. sinensis* (Fig. 1a), and whole shoot tips were collected before bolting (Fig. 1b). Overall, Illumina sequencing produced more than 2.61 billion clean reads (Table 1), while SMRT sequencing generated 526,679 reads. Of the total SMRT reads, 413,886 were full-length non-chimeric (flnc) with an average read length of 2024 bp (Table 2). To reduce the high error rates of the subreads, all SMRT reads were corrected using the cleaned Illumina reads as input (Supplementary Table S1). A total of 43,438 unique transcripts were generated with lengths ranging from 89 to 11,414 bp, with an average of 1996 bp (Fig. 2). The polymerase reads and subreads are presented in Supplementary Table S2. The results showed that SMRT generated high-quality transcripts in *A. sinensis*.

**Table 1:** *A. sinensis* NGS data.

Item	BP	NP
Raw reads number (million)	135.4	135.3
Clean reads number	133.1	132.1
Clean reads rate (%)	98.25	97.66
Raw bases number (Gb)	6.7	6.7
Clean bases number	6.6	6.6

*BP* Bolting plants, *NP* normal plants.

^a^Reads with a quality score < 30 and length < 60 bp were excluded.

**Table 2:** Statistics of SMRT sequencing data.

Library	Number
Polymerase reads	722,134
Number of reads of insert	357,828
Number of full-length non-chimeric reads	413,886
Average of full-length non-chimeric read length	2,024

### Functional annotation and classification of genes

Gene annotation was performed by aligning the 43,438 full-length transcripts generated by SMRT sequencing to the following public databases: the NCBI non-redundant protein (NR) database; the Uni-Prot Knowledgebase (https://www.uniprot.org/); the EuKaryotic Ortholog Groups (KOG) database; the Pfam database (http://pfam.-sanger.ac.uk/); the KEGG Ortholog database (KO) and Gene Ontology (GO) databases by BLASTX; and the NCBI nucleotide sequences (NT) database by BLASTN (E-value ≤ 1e^−5^). Approximately 98.87% of the total genes identified from SMRT sequencing were successfully annotated using these databases (Table 3), of which 12,537 were simultaneously annotated by the NR, NT, BLASTX, and BLASTP databases (Supplementary Fig. S1). A total of 11,469 genes showed significant homologies to genes distributed across 25 categories in the KOG database (Supplementary Fig. S2).

**Table 3:** *A. sinensis* nucleotide database alignment.

Data base	Value	Percentage (%)
NR	22,059	97
NT	17,662	77
BLASTX	19,481	85
BLASTP	17,665	77
Pfam	16,603	72
eggNOG	15,630	68
KO	8640	37

For functional classification, genes were mapped onto the GO database (http://www.geneontology.org/). In total, 22,169 genes were classified into three overarching categories, namely, biological processes, cellular components, and molecular functions (Supplementary Fig. S3). Transcription factors (TFs) regulates many morphology and biological processes, hence their identification is of interest in the sequencing data. The transcriptomic data revealed 19,164 putative genes encoding TFs, which were classified into 60 gene families within the Plant TFDB 5.0 (http://planttfdb.gao-lab.org) (Supplementary Fig. S4). The top ten families with the highest representation were the basic/helix-loop-helix (bHLH), a novel MYB-like gene (MYB-related), NAM, ATAF, and CUC (NAC), WRKY, B3-like DNA binding domain (B3), FAR-RED IMPAIRED RESPONSE 1 (FAR1), Cys3His zinc finger domain (C3H), zinc finger sequence CX2-4CX3FX5LX2HX3-5H (C2H2), basic-leucine zipper (bZIP), and Ethylene-responsive factor (ERF) TF families, respectively. The identification of these TFs will allow a better understanding of the regulation of gene expression that underlines the bolting process in *A. sinensis*.

### Differentially expressed genes (DEGs)

To evaluate differential gene expression levels in response to early bolting, two groups of bolting plants (BP) and non-bolting (normal) plants (NP) Illumina clean reads were taken to assemble with the SMRT full-length transcriptome (Supplementary Table S3). Fragments per kilobase per million reads (FPKM) values of assembling unigenes were calculated with |log_2_ratio|≥ 1 and *P* < 0.05. In summary, 475 DEGs between the BP and NP groups were identified, of which 208 genes were up-regulated, while 267 genes were down-regulated between the two groups. The corresponding genes hierarchical clustering thermogram was showed in Supplementary Fig. S5.

**Figure 3:**
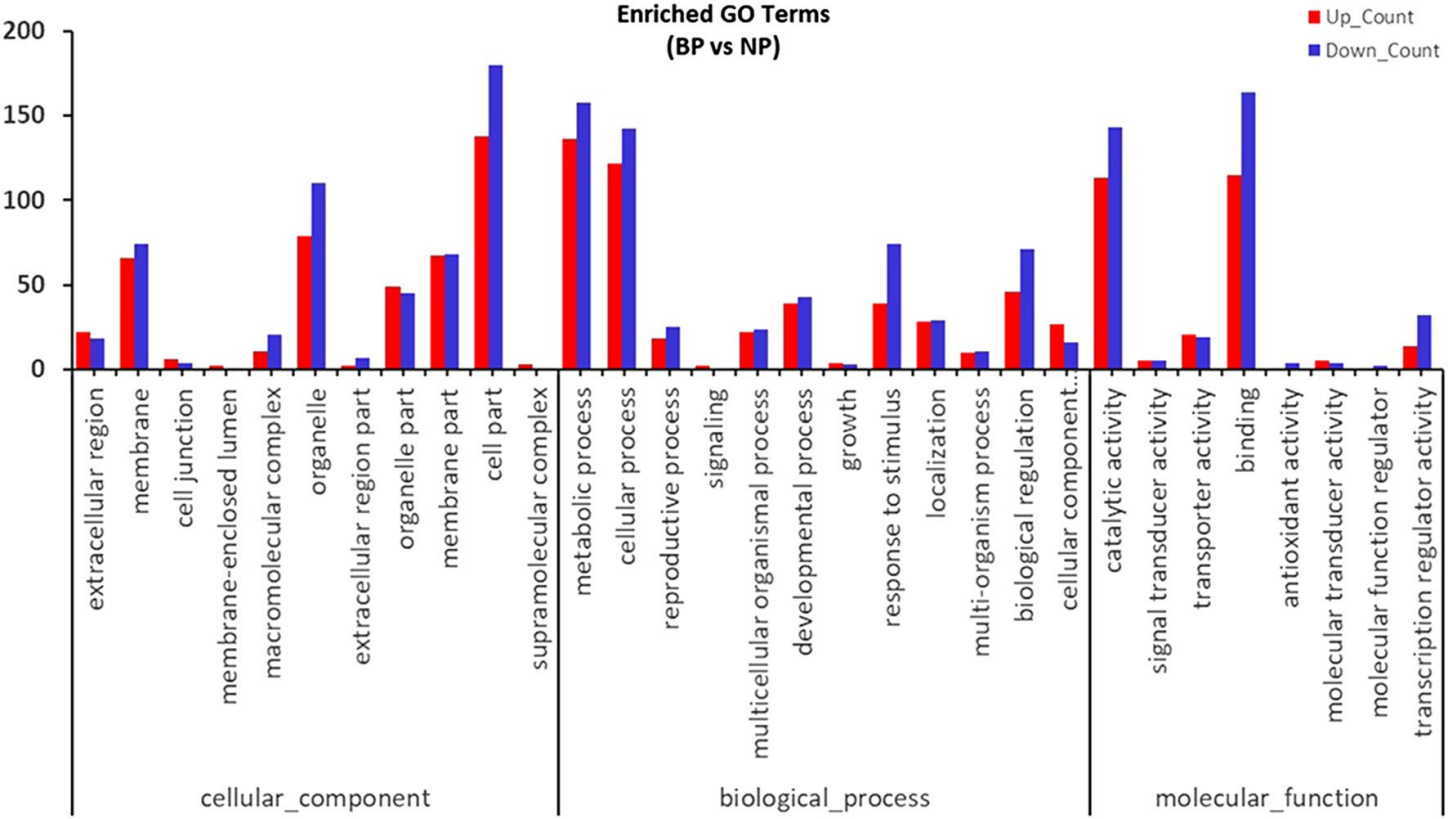
Enriched GO Terms of DEGs generated from the bolting plant (BP) and normal plant (NP) group.

Functional enrichment analysis was conducted to determine the biological functions of the DEGs. A total of 475 DEGs were classified into 42 functional groups using GO assignments (Fig. 3) as 18 functional groups were involved in biological processes, 14 in cellular components, and 10 are in molecular functions. Within the biological process groups the functional groups with the largest enrichment were “metabolic process” containing 136 DEGs (59.65%), “cellular process” containing 122 DEGs (53.51%), “biological regulation” containing 46 DEGs (20.18%), and “response to stimulus” containing 39 DEGs (17.11%). In the two largest functional groups within “molecular function” processes, 115 DEGs (50.44%) were assigned to “binding”, and 113 DEGs (49.56%) were assigned to “catalytic activity”. For the “cellular component” domain, approximately 61% of DEGs (138 total) were assigned to “cell part”, while 34.65% (79 DEGs), 29.39% (67 DEGs), and 28.95% (66 DEGs) were assigned to “organelle,” “membrane part,” and “membrane”, respectively.

Furthermore, 475 DEGs were successfully annotated to 133 KEGG pathways to further characterize the molecular functions and biological pathways. A KEGG scatter plot was shown in Supplementary Fig. S6. In conclusion, these results provide insight into the regulatory elements of *A. sinensis*, which participate in the early bolting process and will contribute to the decoding of these genes.

### DEGs associated with early bolting

**Figure 4:**
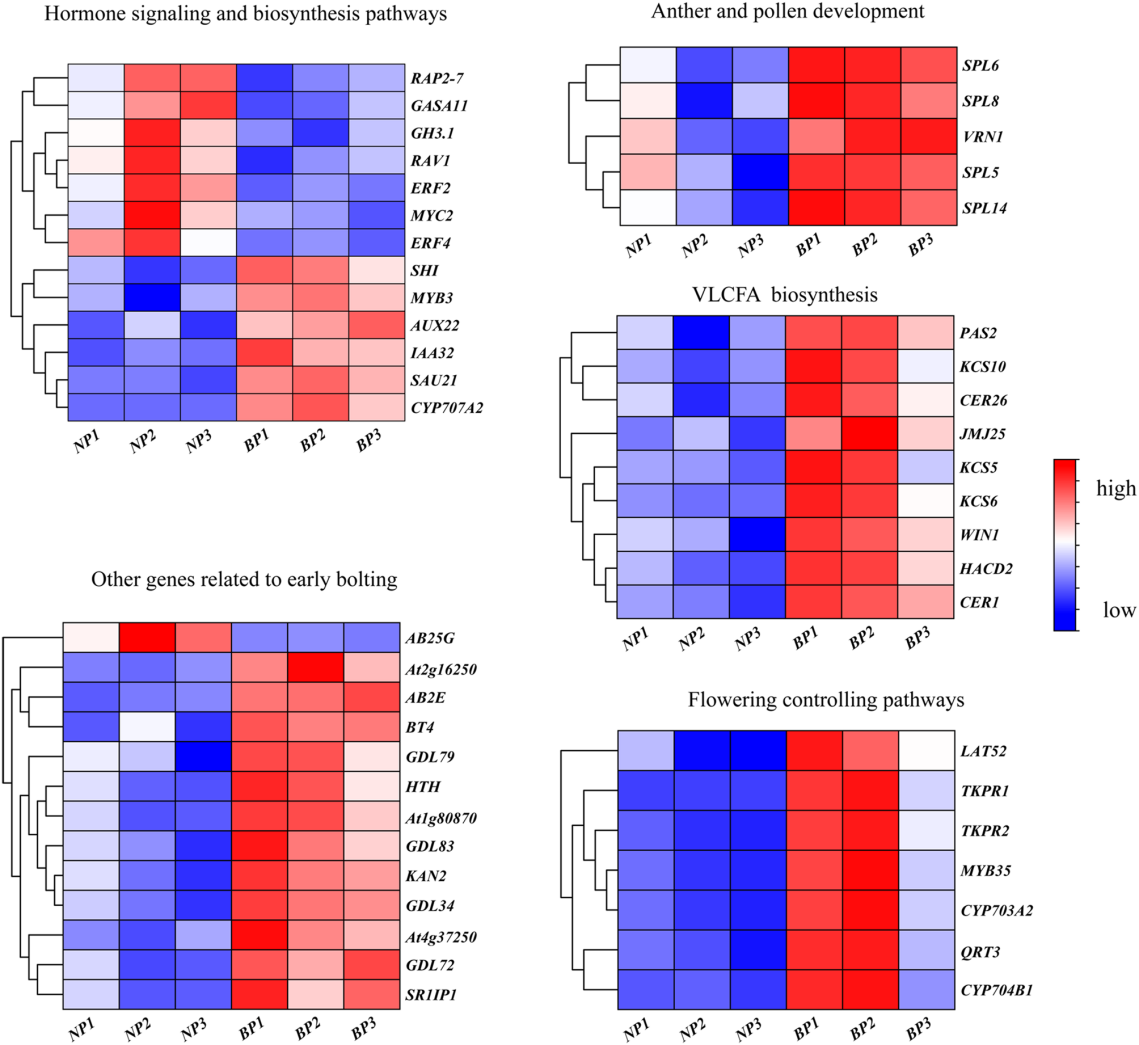
Heatmap of DEGs putatively involved in early bolting between BP and NP. Differential gene expression is based on FPKM values. Gene comparison table from *A. sinensis* to *Arabidopsis thaliana* are provided in Supplementary Table S6.

Several DEGs in biochemical and physiological pathways are known to be associated with bolting and flower were identified (Supplementary Table S4). Genes involved in plant hormone signaling pathways, including Auxin/Indole-3-acetic acid genes (*AUX22*, *IAA32*), Small Auxin Up RNA 21 (*SAUR21*), and Shot Internods gene (*SHI1*) were found to be up-regulated in BP*,* whereas Gretchen Hagen 3 (*GH3.1*)^19–25^, Ethylene-Responsive element-binding Factors (*ERF4*, *ERF99*), for Related to ABI3/VP1 (*RAV1*), microRNA and its APETALA2-Like target gene (*RAP2-7*)^26–28^*,* Myelocytomatosis genes (*MYC2*), and GA-Stimulated in Arabidopsis 11 (*GASA11*) were all down-regulated^29–32^. Genes involved in hormone synthesis pathways, such as 9-cis-epoxycarotenoid dioxygenase (*NCED1*)*,* Cytochrome P450 707a gene family (*ABAH2, ABAH4*), and 4-Coumarate-CoA ligase-like 1 (*4CLL1*) were found to up-regulated, Cytokinin oxidase/dehydrogenase 7 (*CKX7*)^33–36^ was down-regulated. Similarly, genes related to three main flowering controlling pathways, include Squamosa Promoter-Binding protein-like genes (*SPL5, SPL6, SPL8, and SPL14*)^37,38^ were up-regulated in BP. Genes associated with early pollen formation were also found up-regulated in BP, such as Tapetal Development and Function 1 (*MYB35*), Cytochrome P450 gene family genes (*CYP704B1, CYP703A2, CYP86A22*), *At4g20050* (*QRT3*), Anther-specific protein coding genes (*LAT52*), and Tetraketide a-Pyrone Reductase genes (*TKPR1*, *TKPR2*)^39–44^. Genes involved in very-long-chain fatty acids (VLCFAs) biosynthesis pathway including Wax Inducer1 (*WIN1*), Very-long-chain (3R)-3-hydroxyacyl-CoA dehydratase PASTICCINO 2 (*PAS2*), 3-Hydroxyacyl-CoA Dehydratase 2 (*HACD2*), Eceriferum genes (*CER1*, *CER26*), 3-Ketoacyl-CoA Synthase genes (*KCS5*, *KCS6*, *and KCS10*), and Glycerol-3-Phosphate Acyltransferase (*GPAT4*, *GPAT6*)^45–49^ were up-regulated. Gene expression analysis confirmed that the expression of the majority of the aforementioned genes was significantly altered in BP as compared to NP group (Fig. 4).

### qRT-PCR validation of genes related to early bolting in *A. sinensis*

**Figure 5:**
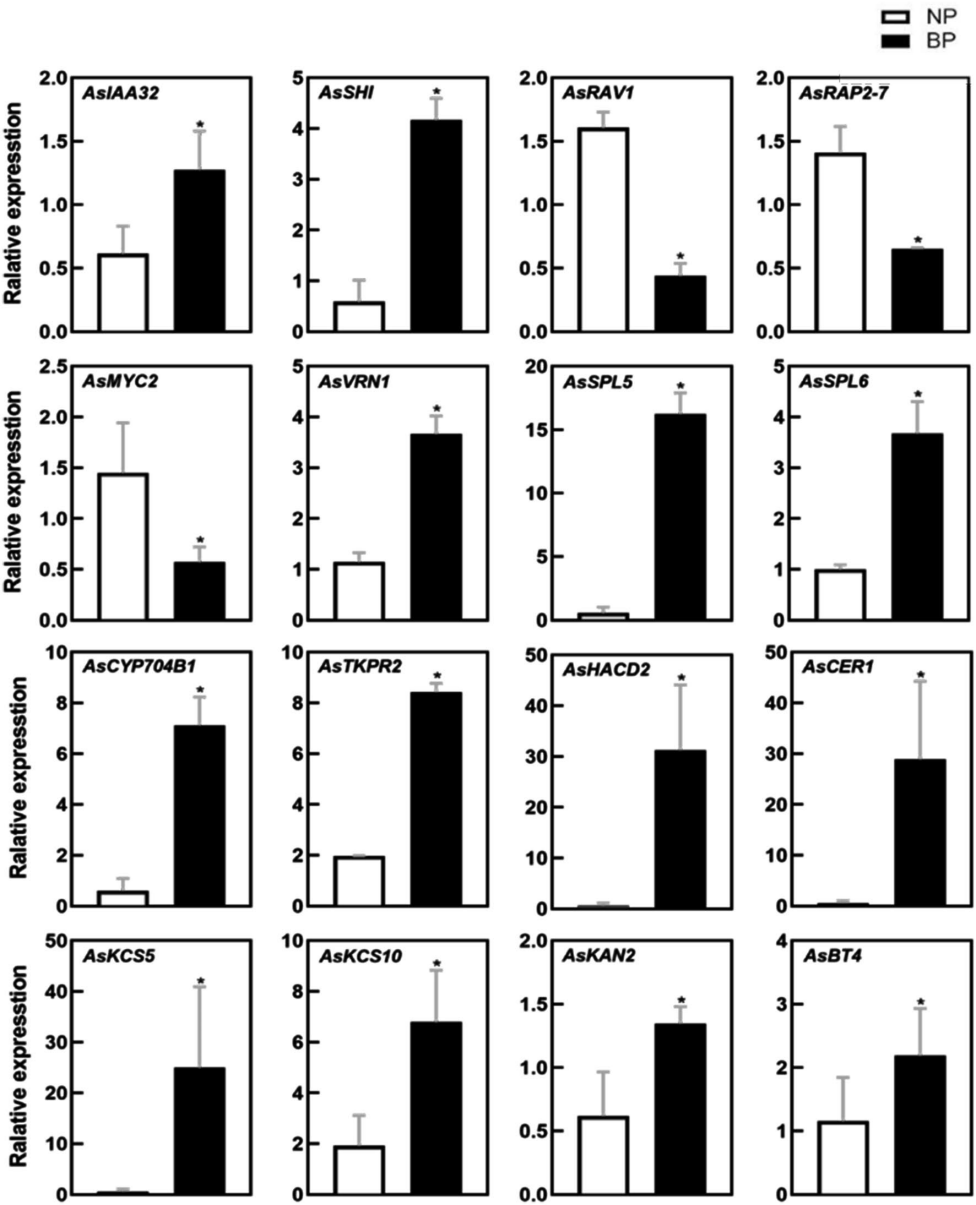
qRT-PCR determined the 16 genes with differential expression between *A. sinensis* bolting plants (BP) and normal plants (NP) grown in the natural environment. All the data represent the values relative to NP as control. * Means differed significantly (*P* < 0.05). Relative expression levels were calculated using the 2^−ΔΔCT^ method.

16 candidate DEGs that were presumably related to early bolting were randomly selected for qRT-PCR analysis to validate the transcriptome data. The differential expression of each of these 16 genes between BP and NP was consistent with transcriptome data (Fig. 5), which confirmed the reliability of the gene expression values obtained from SMRT and NGS sequencing.

### Endogenous hormone contents in early bolting *A. sinensis*

**Figure 6:**
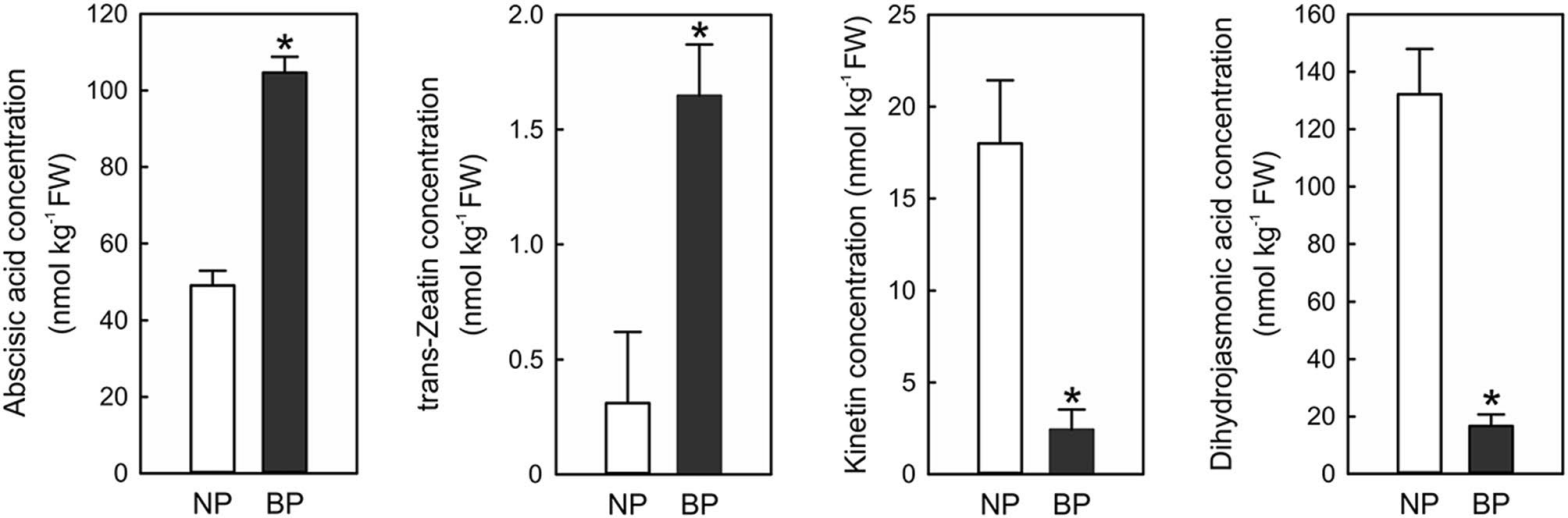
Endogenous hormonal levels change of *A. sinensis* between BP and NP before bolting.

Abscisic acid (ABA), cytokinins (CKs), and jasmonic acid (JA) content were found to change significantly in BP. As shown in Fig. 6, ABA content in BP was significantly higher than NP. Also, two active forms of CKs, including kinetin and trans-Zeatin levels have changed dramatically. For instance, kinetin levels in BP are lower than NP, while trans-Zeatin levels are higher than NP. Besides, the synthesis of dihydro-jasmonic acid was significantly decreased in BP.

## Discussion

*A. sinensis* has a long history of use as a traditional herbal medicine in China, however the early bolting of *A. sinensis* severely restricted its sustainability of resource utilization. Early bolting greatly reduced the accumulation of secondary metabolites contents like ferulic acid and soluble sugar in the roots of *A. sinensis*^50^, causing a complete loss in its medicinal value. Moreover, the genetic background of *A. sinensis* is still unclear*,* which further limits research on its cultivar improvement. Recently, high-throughput sequencing technology especially NGS has been widely used to generate large amounts of omics data of medicinal plants, however, a major limitation of NGS is the length of the short reads, which affected the accuracy of sequence assembly^51^. Single-molecule long-read sequencing offers full-length reads that reduce mis assembles of genes with high sequence identity, greatly improving the accuracy of de novo transcriptome assembly^52^. Therefore, a hybrid sequencing approach combining both short and long-read sequencing technologies provides high-quality and more accurate assemblies for transcriptomic studies in non-model species^53,54^. In the present study, a valid utility was demonstrated based on a hybrid SMRT and NGS sequencing approach for determining the early bolting molecular mechanism of the alpine perennial medicinal plant *A. sinensis*. Overall, the research outcomes increase the understanding of early bolting in *A. sinensis* at the molecular level, and also provides complete transcriptome resource for *A. sinensis*. In future, this knowledge could be applied in the selection of high bolting-tolerant germplasm resources and molecular breeding of *A. sinensis* to develop bolting-tolerant *A. sinensis* varieties for the traditional Chinese medicine market.

Plant hormones regulate multiple aspects of growth and development, including flowering time. Among the DEGs in the plant hormone signaling pathways, many genes related to auxin signaling (Fig. 4; Supplementary Table S4), such as *IAA32*, *AUX22*, *SAUR21*, and the auxin response transcription activator, *SHI1* were up-regulated in BP; concurrently, the negative regulator of auxin response *GH3.1* was down-regulated^19–22^. Previous studies have revealed that auxin and its corresponding receptors are necessary for the initiation of flowering and floral organ identity^23,24^. It is worth noting that *SHI* regulates flowering time and promotes pistil development^25^, signifying its key role in promoting early bolting. Four ethylene response transcription factors (ERFs), namely *RAV1*, *ERF4*, *ERF99*, and *RAP2-7* were down-regulated in BP. Moreover, it was earlier reported that ERFs are involved in the regulation of Arabidopsis bolting^26^. Down-regulation of *RAV1* in Arabidopsis leads to an early flowering phenotype^27^. *RAP2-7* negatively regulates the transition from vegetative to reproductive growth, results in a delay in flowering time^28^. The down-regulated expression of *RAV1* and *RAP2-7* in BP is therefore consistent with its early bolting phenotype. In the JA signaling pathway, the transcription factor *MYC2*^29^ was down-regulated in BP. *MYC2* is a member of the basic helix-loop-helix transcription factor family and is a high-level transcription regulatory element in the JA signaling pathway and has been shown to participate in JA-mediated flowering inhibition in Arabidopsis^30,31^. Finally, GA signal response gene *GASA11*, which putatively contribute to hormone-regulated flowering was also down-regulated in BP^32^.

To validate the transcriptome analysis and further explore the effect of hormones on early bolting and flowering of *A. sinensis*, a total of 24 hormones in BP and NP were identified. UHPLC results showed the level of endogenous hormones, including ABA, JA, and CKs in BP were significantly changed. Genes responsible for the biosynthesis/metabolism of these endogenous hormones in BP, including ABA synthesis genes *NCED1*^33^, metabolic genes *ABAH2* and *ABAH4*^34^, and JA synthesis genes *4CLL1*^35^ were up-regulated, whereas CTK synthesis genes *ZOG* and *CKX7* were down-regulated^36^. Altogether, genes involved in multiple hormones signaling or biosynthesis/metabolism pathways regulate early bolting of *A. sinensis* were differentially expressed between the two phenotypes, suggesting early bolting was simultaneously controlled by multiple hormones. These findings also revealed that bolting and flowering in *A. sinensis* were regulated by the complex genetic network.

Members of the square promoter binding protein-like (SPL) family of transcription factors, including *SPL5*, *SPL6*, *SPL8*, and *SPL14* were also up-regulated at different levels in BP, hence attracted our special attention. SPL genes regulate flowering through the photoperiod pathway and can directly activate specific genes like *LFY*, *FUL*, and *AP1* to further promote flowering through the aging pathway, which is dependent on endogenous miRNA level (miR156 and miR172)^37,38^. The relationship between environment and bolting as well as genes that are directly or indirectly involved in this regulatory network, including SPL, warrant further investigation to determine their role in *A. sinensis* early bolting.

We noticed that a large number of genes related to pollen formation were up-regulated in BP, including *MYB35*(*TDF1*), *TKPR1* and *TKPR2, QRT3, LAT52, CYP704B1*, *CYP703A2,* and *CYP86A22* (Fig. 4; Supplementary Table S4). *MYB35*, an R2R3 MYB transcription factor was previously identified in *Arabidopsis* as a putative transcription factor regulator of tapetal development and function^39^. The *Arabidopsis* CYP450 family, *TKPR1* and *TKPR2* are conserved genes in land plants that control the production of sporopollenin, a major constituent of the exine of pollen^40^. Whereas *CYP704B1* and *CYP703A2*, *CYP86A22*, *QRT3*, and *LAT52* are all key factors in both pollen and anther development^41–44^, *TKPR1* and *TKPR2* are associated with the early stages of anther development. The up-regulated expression of multiple genes related to pollen formation suggests that pollen production is initiated in anticipation of the plant transaction from its vegetative phase to its reproductive phase.

Interestingly, in the present study genes involved in the regulation of the cuticle and epidermal wax production are relatively up-regulated. The expression of the ethylene response factor *WIN1, PAS2, HACD2, CER1* and *CER26, KCS5, KCS6, KCS10, GPAT4*, and *GPAT6* were up-regulated in BP (Fig. 4; Supplementary Table S4). The majority of these genes are involved in the biosynthesis of very-long-chain fatty acids (VLCFA)^45–47^. VLCFAs are direct precursors of wax compounds that are synthesized in the epidermis. They are essential for plant development, and have been reported for their involvement in cellular communications, mainly in pollen-stigma interactions^48^. Recent studies have shown that VLCFAs may regulate cell proliferation in the Arabidopsis shoot apex^49^. These genes were assigned to six main KEGG pathways which centered around fatty acid biosynthesis and metabolism (Supplementary Fig. S6)^55^. The up-regulation of genes that promoted VLCFAs synthesis in accordance with those genes related to pollen development suggests an imminent transition to flowering in BP. In another words, this may also accelerate the transformation of vegetative to reproductive growth phase, and consequently triggered early bolting. Further investigation on the functions of VLCFAs related to flowering may identify useful targets for the development of slow bolting *A. sinensis* varieties.

The expression of genes controlling cell differentiation was also differentially expressed in the two genotypes, namely KANADI2 (*KAN2*) and HOTHEAD (*HTH*) (Fig. 4; Supplementary Table S4). The functions of *KAN2* and *HTH* were reported in determining the fate of paraxial stem cells, and in maintaining the activity of the shoot apical meristem^56,57^. The stems of *kan2* mutants failed to elongate during flowering, which was consistent with the phenotype of BP in Min County. Plants require additional energy when entering the reproductive growth phase to sustain reproduction, and therefore genes related to energy acquisition may be up-regulated. Indeed, genes associated with energy transport, ubiquitination, and enzyme-mediated reactions were found to be up-regulated in BP, including ABC transporter G family member25 (*ABCG25*), GDSL esterase/lipase At5g03810 (*GDL72*), and E3 ubiquitin-protein ligase complex encoding gene *BT4*^58,59^.

**Figure 7:**
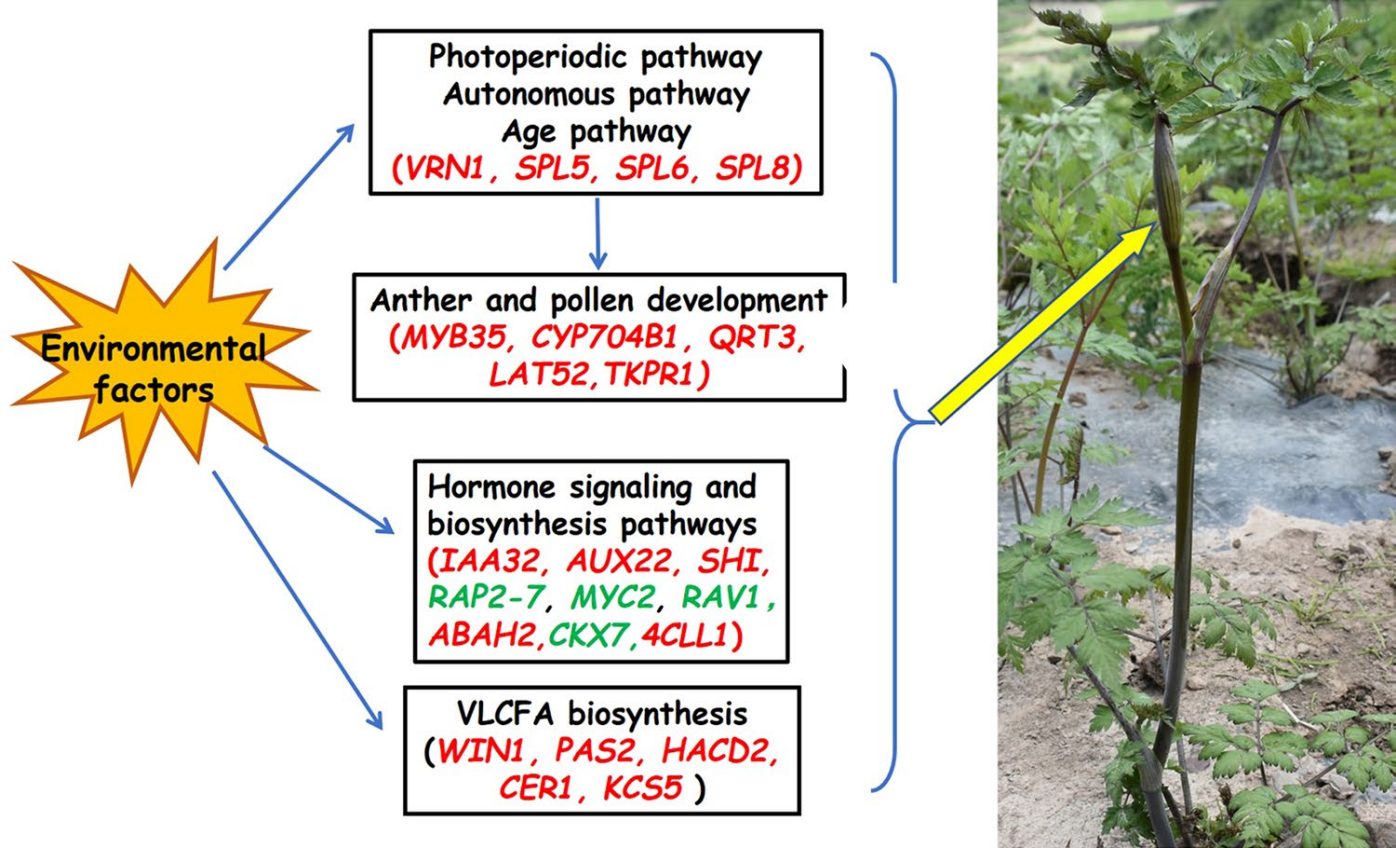
The putative genes interaction model for early bolting of *A. sinensis* in the natural environment. The early bolting process of *A. sinensis* involves the transformation of different development patterns, the development of flower organs, and the interaction of signal pathways controlled by the environment and another endogenous factor.

Various signaling factors of bolting induction pathways regulated the expression of a group of meristem-specific genes that determines the growth characteristics of *A. sinensis*. A model of DEGs involved in early bolting and their potential interactions is presented in Fig. 7. Overall, our findings offer comprehensive information on *A. sinensis* transcriptome analysis, which could be further used to develop early bolting resistance varieties, and subsequently supports a higher yield of medicinally important *A. sinensis* roots.

## Methods

### Plant materials and growing conditions

*A. sinensis* seeds were collected on July 26 in the year 2016 from the 3-year seed plants. It was sowed on June 1 in the year 2017 for cultivation of seedling at the study site in Min County, Gansu province, P. R. China (N 3425′ 7″, E 10,428′ 24″, elevation 2783 m). The site of cultivation is mountainous with meadow soil and a rainfed agroecosystem. It has a cool and semi-humid climate with an annual average temperature of 5–6 °C, approximately 2219 h annual sunshine, 90–120 frost-free days per year, and 451.4–817.8 mm of annual rainfall, mainly from June to September^60^.

*A. sinensis* seedlings were transplanted and cultivated on April 20 in the year 2018 in another study site on the same mountain in Min County, China. Whole shoot tips and young developing leaves from actively growing shoots of 6 typical *A. sinensis* individual plants were collected before bolting in July 2018. These were immediately frozen in liquid nitrogen, and stored at − 80 °C until RNA extraction. Normal plants (NP) that were not bolting served as the control group, while bolting plants (BP) were the experimental group. Each group consisted of three different plants representing three biological replicates. A single shoot tip was harvested and analyzed for each replicate. The medicinal plant was identified by Prof. Yuan Chen (Gansu Agriculture University, China). The plant sample collection did not require any specific permission and were deposited in the specimen room of Gansu Agriculture University, China.

### RNA isolation and NGS

High-quality RNA samples were extracted using Trizol reagent (Invitrogen, CA, USA) and treated with RNase-free Dnase (Takara, Dalian, China). Sequencing libraries were constructed using the NEBNext® Ultra™ RNA Library Prep Kit for Illumina® (New England Biolabs). Next-generation sequencing (NGS) was performed by Annoroad Gene Technology (Beijing, China) using the Illumina HiSeq X Ten platform in high output mode to produce 150 bp paired-end reads.

### PacBio Iso-Seq library preparation and SMRT

Libraries for SMRT sequencing were generating by pooling equal amounts of the six RNA samples as previously described. The libraries preparation process was carried out according to the Iso-Seq protocol by Pacific Biosciences (P/N 101-070-200 Version 06). First strand cDNA synthesis employs the SMARTer PCR cDNA Synthesis Kit (Clontech 634926), and the resultant cDNA was amplified using KAPA HiFi PCR Kits (Pacific Biosciences, CA, USA). Amplified cDNA was fractionated into > 4 kb fragments by Blue Pippin Size Selection v5.90 (Sage Science, Beverly, MA, USA) and subsequently used to construct one library of different insert sizes using the SMRTbell™ Template Prep Kit v1.0 (Pacific Biosciences, USA). Finally, after quantification, the library templates and enzyme complexes (V2 reagents, PacBio) with a certain concentration and volume were transferred to the nanopores of the PacBio Sequel sequencing instrument for sequencing.

### Iso-Seq data processing with standard bioinformatics pipeline

Polymerase reads (raw sequencing data) were processed using the SMRTlink software (v7.0) with the following parameters: –min Length 50, –max Length 15,000, –min Passes 1^61^. Reads of Insert (ROI) from polymerase reads after the detection and removal of polyA tail by cDNA primers were separated into full-length, non-chimeric reads, and non-full length reads. Full-length non-chimeric ROIs were clustered and assembled into consensus isoforms by ICE (isoform-level clustering algorithm). Finally, high quality isoforms HQ (above 99% accuracy) and low-quality isoforms LQ were obtained after polished by Quiver.

### Functional annotation and classification

The annotation of genes function was used Trinotate (20,140,717) by the following database: NT (NCBI non-redundant nucleotide sequences, cutoff E-value ≤ 1e^−5^), NR (NCBI non-redundant protein sequences), Uni-Prot (https://www.uniprot.org/), SingalP (http://www.cbs.dtu.dk/services/SignalP/), PFAM (http://pfam.xfam.org/), eggNOG (http://eggnog5.embl.de), KOG (euKaryotic Ortholog Groups), KO (KEGG Ortholog database) and GO (Gene Ontology, http://www.geneontology.org/). The Blast2GO program (http://www.blast2go.com) was used to annotate GO terms. The prediction of transcription factors (TFs) in *A. sinensis* was conducted by using the database PlantTFDB 5.0 (http://planttfdb.gao-lab.org/).

### Gene expression levels quantification

NGS data were compared with the reference sequence using the software package RSEM (v1.3.1). We compare the read-count of each isoform in each sample, and convert the FPKM value to obtain the expression level of each isoform. FPKM considering both the depth of sequencing and the influence of isoform length on fragments is a general method for estimating isoforms expression level.

### Analysis of differentially expressed genes (DEGs)

The DEGSeq R package (v1.28.0) was used to identify DEGs between the BP and NP samples^62^. Clustering patterns of DEGs between BP and NP were determined by the Euclidean distance cluster analysis method, and heatmaps were drawn by pheatmap R package (v1.0.12)^63^. Genes with an absolute log2 (BP/NP) value ≥ 1 and *P* values ≤ 0.05 were identified as significant DEGs. GO functional enrichment analysis of the DEGs was performed using the GOseq R package (v1.26.0) based on the Wallenius non-central hyper-geometric distribution^64^. The software KOBAS (v2.0) was used to test the statistical enrichment of DEGs in KEGG pathways^65^. After multiple testing corrections, a KEGG scatter plot was drawn by adjusted *P* value ≤ 0.05.

### Validation of DEGs using qRT-PCR

Quantitative RT-PCR (qRT-PCR) was used to validate 16 candidate DEGs associated with early bolting. The *AsACTIN* gene was used as an internal control^66^. Three technical repeats were used for each gene, and the data shown are representative of three independent experiments. RNA for validation is based on previous sequencing samples. All reactions were performed with the CFX96 Real-Time PCR System (Bio-Rad, CA, USA) using HiScript® II Q RT SuperMix for qPCR (+ gDNA wiper) (Vazyme, Nanjing, China). The primers sequences can be found in Supplementary Table S5.

### Determination of endogenous hormone levels

The endogenous hormonal levels of *A. sinensis* were determined by BIOTREE Biotechnology Co., Ltd in Shanghai. The young developing leaves from actively growing shoots of *A. sinensis* before bolting were grinded into powder in liquid nitrogen and each sample was precisely weighed for 20 mg aliquot, then put into extract solution (50% acetonitrile in water, precooled at − 40 °C, containing isotopically-labelled internal standard mixture), thereafter further purified with SPE^67^. The purified product was then subjected to ultra-high performance liquid chromatography-tandem mass spectrometry (UHPLC-MS/MS) analysis. The UHPLC separation was carried out using an EXIONLC System (Sciex), equipped with a Waters ACQUITY UPLC CSH C18 column (150 × 2.1 mm, 1.7 μm, Waters). Mobile phase A contains 0.01% formic acid in water, and the mobile phase B was 0.01% formic acid in acetonitrile. The column temperature was set at 50℃ and the auto-sampler temperature was set at 4 °C. The injection volume was 5 μL. A SCIEX 6500 QTRAP + triple quadrupole mass spectrometer (Sciex), equipped with an IonDrive Turbo V electrospray ionization (ESI) interface was applied for assay development. Typical ion source parameters were: Curtain Gas set at 40 psi, IonSpray Voltage set at ± 4500 V, the temperature was set at 475 °C, and the Ion Source Gas 1 and 2 was set at 30 psi. SCIEX Analyst Work Station Software (Version 1.6.3) and Sciex MultiQuant™ 3.0.3 were employed for MRM data acquisition and processing.

## Supplementary Information

Supplementary Figures S1.

Supplementary Figures S2.

Supplementary Figures S3.

Supplementary Figures S4.

Supplementary Figures S5.

Supplementary Figure S6.

Supplementary Table S1.

Supplementary Table S2.

Supplementary Table S3.

Supplementary Table S4.

Supplementary Table S5.

Supplementary Table S6.
