# Supplementary material for: Full-length transcriptome analysis provides new insights into the early bolting occurrence in medicinal Angelica sinensis
Source: Sci Rep. 2021 Jun 21;11:13000. doi: 10.1038/s41598-021-92494-4 (PMC8217430; doi:10.1038/s41598-021-92494-4)
Supplement: Supplementary file 1 — Supplementary Figures S1. [file 41598_2021_92494_MOESM1_ESM.docx]

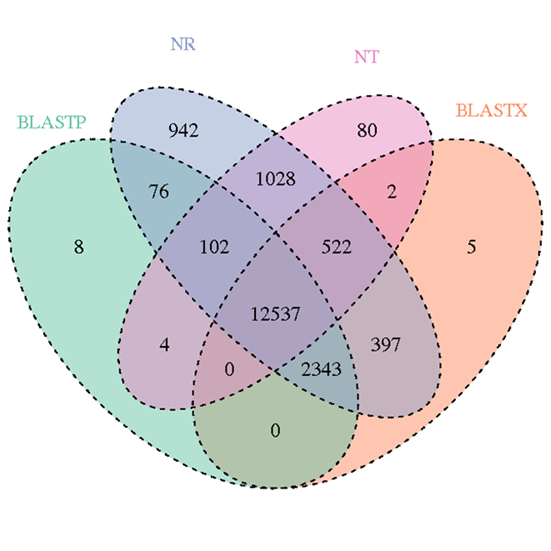


**Supplementary Fig. S1** Venn diagrams describing the numbers of unique and shared differentially expressed genes (DEGs) between samples
