# Supplementary figures and images for: Full-length transcriptome analysis provides new insights into the early bolting occurrence in medicinal Angelica sinensis
Source: Sci Rep. 2021 Jun 21;11:13000. doi: 10.1038/s41598-021-92494-4 (PMC8217430; doi:10.1038/s41598-021-92494-4)

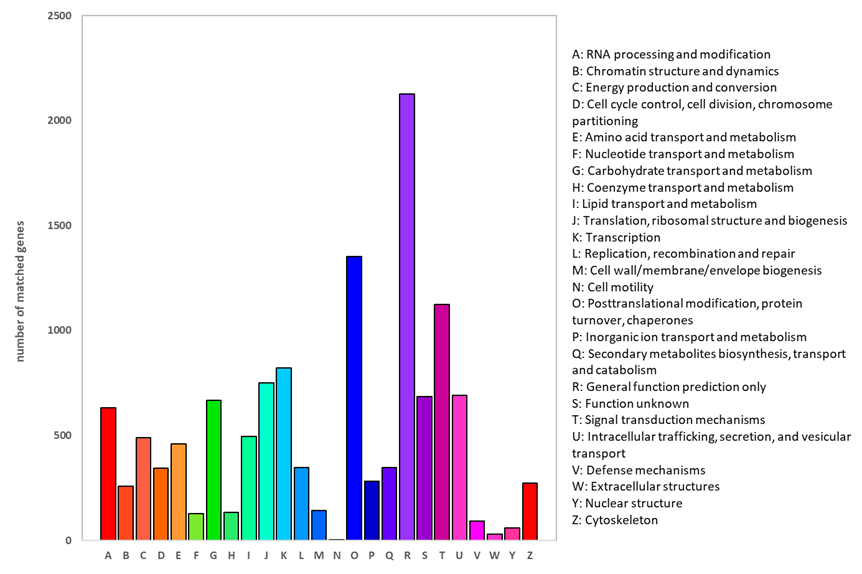


**Supplementary Fig. S2** KOG function annotation

Supplement: Supplementary file 2 — Supplementary Figures S2. [file 41598_2021_92494_MOESM2_ESM.docx]

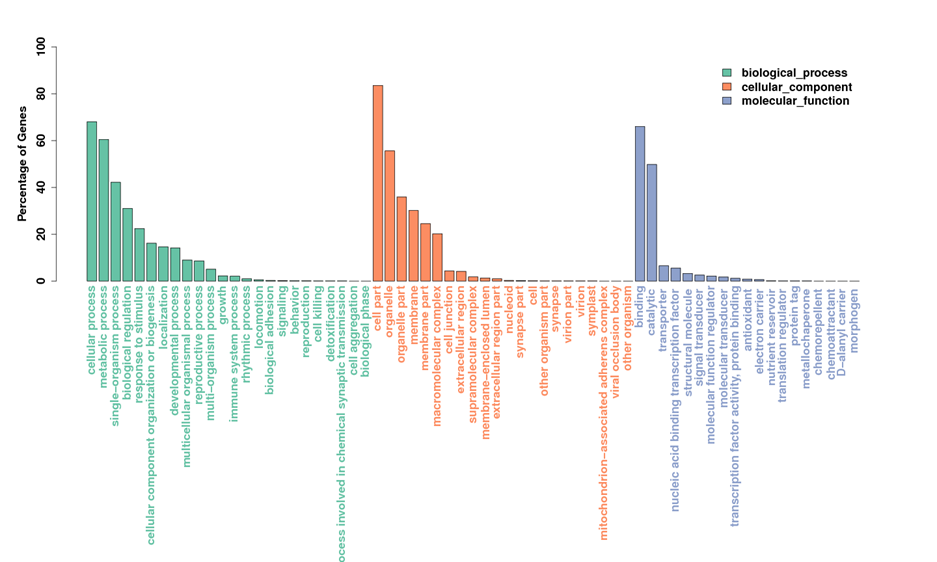


**Supplementary Fig. S3** SMRT GO Classification

Supplement: Supplementary file 3 — Supplementary Figures S3. [file 41598_2021_92494_MOESM3_ESM.docx]

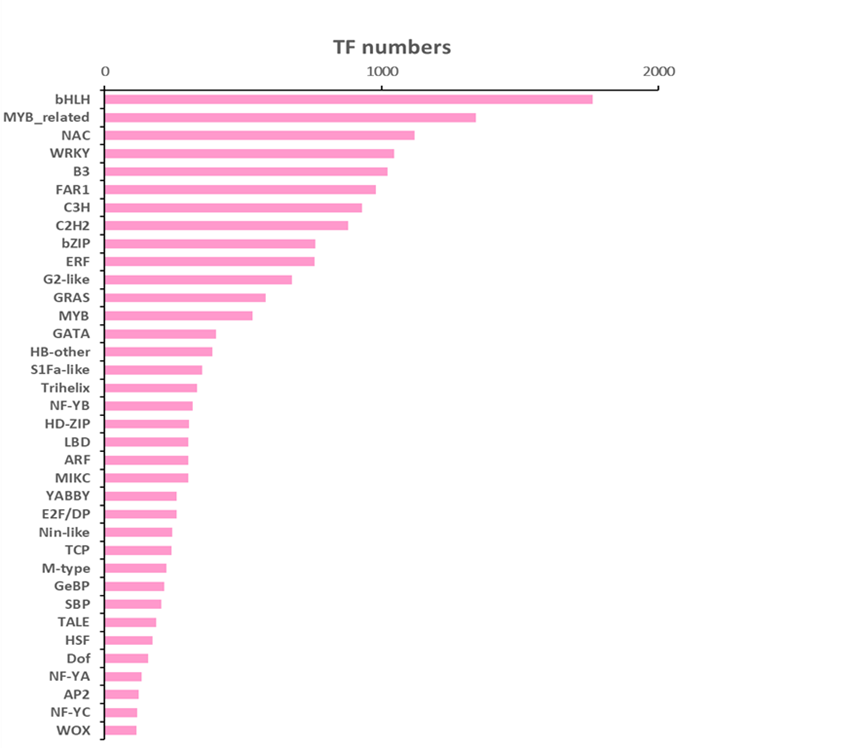


**Supplementary Fig. S4**  Top 36 TFs families predicted by SMRT

Supplement: Supplementary file 4 — Supplementary Figures S4. [file 41598_2021_92494_MOESM4_ESM.docx]

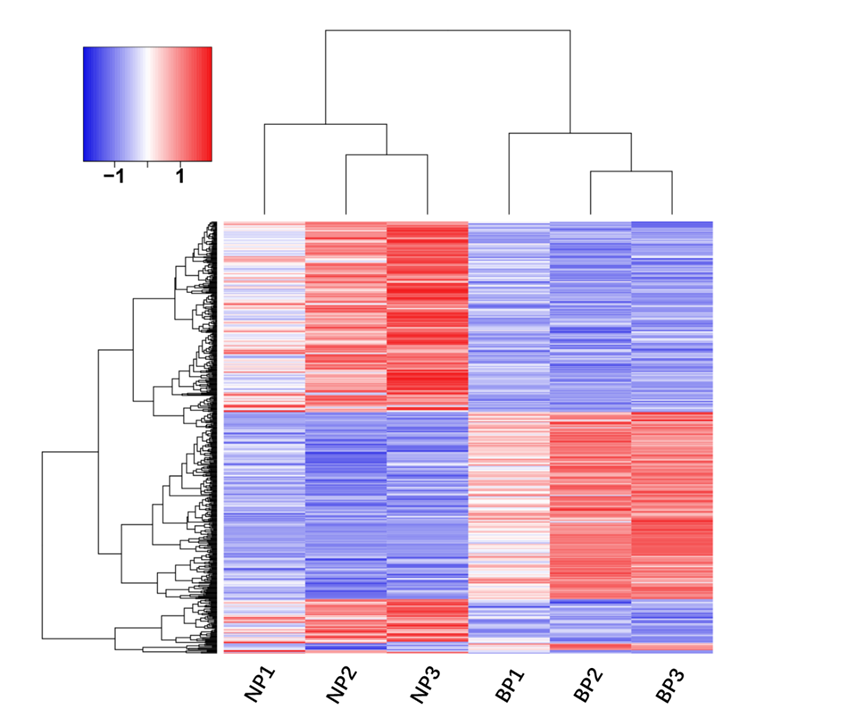


**Supplementary Fig. S5** Clustering analysis of the DEGs

Supplement: Supplementary file 5 — Supplementary Figures S5. [file 41598_2021_92494_MOESM5_ESM.docx]

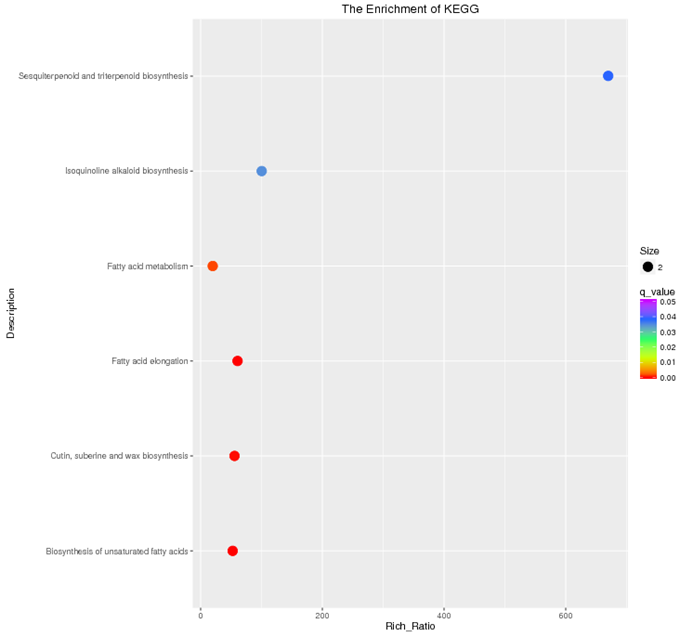


**Supplementary Fig. S6** KEGG enrichment of DEGs

Supplement: Supplementary file 6 — Supplementary Figure S6. [file 41598_2021_92494_MOESM6_ESM.docx]
