# Supplementary material for: Full-length transcriptome analysis provides new insights into the early bolting occurrence in medicinal Angelica sinensis
Source: Sci Rep. 2021 Jun 21;11:13000. doi: 10.1038/s41598-021-92494-4 (PMC8217430; doi:10.1038/s41598-021-92494-4)
Supplement: Supplementary file 7 — Supplementary Table S1. [file 41598_2021_92494_MOESM7_ESM.docx]

| Sample | NP1 | NP2 | NP3 | BP1 | BP2 | BP3 |
| --- | --- | --- | --- | --- | --- | --- |
| Raw Reads Number | 47,866,634 | 45,618,220 | 41,797,164 | 44,432,524 | 47,808,402 | 43,181,294 |
| Raw Bases Number | 7,18G | 6,84G | 6,27G | 6,66G | 7,17G | 6,48G |
| Clean Reads Number | 46,752,576 | 44,691,124 | 40,689,584 | 43,571,466 | 46,981,832 | 42,501,038 |
| Clean Reads Rate (%) | 97.67 | 97.97 | 97.35 | 98.06 | 98.27 | 98.42 |
| Low-quality Reads Number | 264,620 | 227,830 | 217,440 | 237,538 | 252,596 | 241,876 |
| Low-quality Reads Rate (%) | 0.55 | 0.5 | 0.52 | 0.54 | 0.53 | 0.56 |
| PolyG Reads Number | 72046 | 62282 | 58146 | 61838 | 65662 | 64802 |
| PolyG Reads Rate (%) | 0.15 | 0.14 | 0.14 | 0.14 | 0.14 | 0.15 |
| Raw Q30 Bases Rate (%) | 94.48 | 94.24 | 94.34 | 93.98 | 94.16 | 94.02 |
| Clean Q30 Bases Rate (%) | 94.71 | 94.45 | 94.56 | 94.2 | 94.38 | 94.25 |

**Supplementary Table S1** Summary of Illumina sequence data quality
