# Supplementary material for: Full-length transcriptome analysis provides new insights into the early bolting occurrence in medicinal Angelica sinensis
Source: Sci Rep. 2021 Jun 21;11:13000. doi: 10.1038/s41598-021-92494-4 (PMC8217430; doi:10.1038/s41598-021-92494-4)
Supplement: Supplementary file 8 — Supplementary Table S2. [file 41598_2021_92494_MOESM8_ESM.docx]

**Supplementary Table S2** Summary of transcripts from SMRT sequencing

**Polymerase reads stat**

| Cell ID | Polymerase  reads | Polymerase  base | Average  polymerase  length | Polymerase  n50 |
| --- | --- | --- | --- | --- |
| ZW | 722,134 | 28,944,546,023 | 40,081.96 | 77,319 |

**Subreads stat**

| Cell  ID | Subreads  reads | Subreads  base | Average  subreads  length | Accuracy | | Subreads  n50 | GC  mean |
| --- | --- | --- | --- | --- | --- | --- | --- |
| ZW | 17,563,342 | 25,586,903,321 | 1,456.84 | | 0.8 | 2,182 | 0.41 |

**CCS stat**

| Sample  id | Reads  Number | Bases  Number | Mean  Length | Max  Length | GC | N50 | Mean  Pass |
| --- | --- | --- | --- | --- | --- | --- | --- |
| ZW | 526,679 | 1,159,908,524 | 2202.31 | 17,924 | 40.73 | 2,561 | 26.93 |
