# Supplementary material for: Full-length transcriptome analysis provides new insights into the early bolting occurrence in medicinal Angelica sinensis
Source: Sci Rep. 2021 Jun 21;11:13000. doi: 10.1038/s41598-021-92494-4 (PMC8217430; doi:10.1038/s41598-021-92494-4)
Supplement: Supplementary file 9 — Supplementary Table S3. [file 41598_2021_92494_MOESM9_ESM.docx]

**Supplementary Table S3** mapping rate by NGS to SMRT sequcencing

| Items |  | BP1 | BP2 | BP3 | NP1 | NP2 | NP3 |
| --- | --- | --- | --- | --- | --- | --- | --- |
| Total Reads |  | 43571466 | 46981832 | 42501038 | 46752576 | 44691124 | 40689584 |
| Mapped Reads |  | 34348526 | 37445566 | 33882192 | 38325174 | 35256164 | 33225722 |
| Mapping Rate |  | 0.7883261 | 0.7970223 | 0.7972086 | 0.8197446 | 0.7888851 | 0.8165658 |
| UnMapped Reads |  | 9222940 | 9536266 | 8618846 | 8427402 | 9434960 | 7463862 |
| MultiMap Reads |  | 10116262 | 10819988 | 9948586 | 11526964 | 10208796 | 9919650 |
| MultiMap Rate |  | 0.2321763 | 0.2303015 | 0.2340787 | 0.2465525 | 0.2284301 | 0.2437884 |
