# Supplementary material for: Full-length transcriptome analysis provides new insights into the early bolting occurrence in medicinal Angelica sinensis
Source: Sci Rep. 2021 Jun 21;11:13000. doi: 10.1038/s41598-021-92494-4 (PMC8217430; doi:10.1038/s41598-021-92494-4)
Supplement: Supplementary file 10 — Supplementary Table S4. [file 41598_2021_92494_MOESM10_ESM.docx]

**Supplementary Table S4** DEGs regulated bolting time and reproductive growth identified in early bolting plants

| **Gene name** | **Protein name** | **identified** | **putative** | **Log2 Fold Change** |
| --- | --- | --- | --- | --- |
| Genes involved in plant hormone signal pathways | | | | |
| *IAA32* | Auxin-responsive protein IAA32 | √ |  | 1.75 |
| *AUX22* | Auxin-induced protein AUX22 | √ |  | 1.94 |
| *SAU21* | Auxin-responsive protein SAUR21 | √ |  | 2.45 |
| *SRS1* | Protein SHI RELATED SEQUENCE 1 | √ |  | 2.04 |
| *GH3.1* | Probable indole-3-acetic acid-amido synthetase GH3.1 | √ |  | −1.52 |
| *RAV1* | AP2/ERF and B3 domain-containing transcription factor RAV1 | √ |  | −1.70 |
| *ERF4* | Ethylene-responsive transcription factor 4 |  |  | −1.98 |
| *ERF13* | Ethylene-responsive transcription factor 13 |  |  | −2.01 |
| *RAP2-7* | Ethylene-responsive transcription factor RAP2-7 | √ |  | −1.27 |
| *MYC2* | Transcription factor MYC2 | √ |  | −1.45 |
| *CYP707A2* | Abscisic acid 8'-hydroxylase 2 | √ |  | 3.57 |
| *GASA11* | Gibberellin-regulated protein 11 |  | √ | −1.53 |
| Genes involved in plant hormone metabolism pathways | | | | |
| *ABAH2*  Abscisic acid 8'-hydroxylase 2 √ 2.58 | | | | |
| *CKX7* Cytokinin dehydrogenase 7 √ − 0.97 | | | | |
| *4CLL1* 4-coumarate--CoA ligase-like 1 √ 4.13 | | | | |
| Genes involved in 3 main flowering controlling pathways | | | | |
| *VRN1* | B3 domain-containing transcription factor VRN1 | √ |  | 1.37 |
| *At5g66980* | Putative B3 domain-containing protein |  | √ | 2.07 |
| *SPL6* | Squamosa promoter-binding-like protein 6 |  | √ | 1.30 |
| *SPL8* | Squamosa promoter-binding-like protein 8 |  | √ | 1.45 |
| Genes involved in early pollen formation | | | | |
| *MYB35* | Transcription factor MYB35 | √ |  | 2.49 |
| *CYP703A3* | Cytochrome P450 703A2 | √ |  | 2.75 |
| *CYP704B1* | Cytochrome P450 704B1 | √ |  | 2.21 |
| *CYP86A22* | Cytochrome P450 86A22 | √ |  | 1.95 |
| *QRT3* | Polygalacturonase QRT3 | √ |  | 1.81 |
| *LAT52* | Anther-specific protein LAT52 | √ |  | 2.09 |
| *TKPR1* | Tetraketide alpha-pyrone reductase 1 | √ |  | 2.88 |
| *TKPR2* | Tetraketide alpha-pyrone reductase 2 | √ |  | 3.07 |
| Genes involved in VLCFA biosynthesis pathway | | | | |
| *WIN1* | Ethylene-responsive transcription factor WIN1 | √ |  | 1.74 |
| *PAS2* | Very-long-chain (3R)-3-hydroxyacyl-CoA dehydratase PASTICCINO 2 | √ |  | 1.15 |
| *HACD2* | Very-long-chain (3R)-3-hydroxyacyl-CoA dehydratase 2 | √ |  | 2.70 |
| *CER1* | Protein ECERIFERUM 1 | √ |  | 2.86 |
| *CER26* | Protein ECERIFERUM 26 | √ |  | 1.40 |
| *KCS5* | 3-ketoacyl-CoA synthase 5 | √ |  | 2.44 |
| *KCS6* | 3-ketoacyl-CoA synthase 6 | √ |  | 2.52 |
| *GPAT6* | Glycerol-3-phosphate 2-O-acyltransferase 6 | √ |  | 1.69 |
| Other genes putatively involved in early bolting | | | | |
| *ABCG25* | ABC transport G family members 25 |  | √ | −2.62 |
| *At2g04570* | GDSL esterase/lipase At2g04570 |  | √ | 2.46 |
| *GDL72*  *BT4* | GDSL esterase/lipase 72  BTB/POZ and TAZ domain-containing protein 4 |  | √  √ | 1.79  1.19 |
