# Supplementary material for: Full-length transcriptome analysis provides new insights into the early bolting occurrence in medicinal Angelica sinensis
Source: Sci Rep. 2021 Jun 21;11:13000. doi: 10.1038/s41598-021-92494-4 (PMC8217430; doi:10.1038/s41598-021-92494-4)
Supplement: Supplementary file 11 — Supplementary Table S5. [file 41598_2021_92494_MOESM11_ESM.docx]

**Supplementary Table** **S5** Sequences of primer employed in qRT-PCR analysis.

| Gene name | Primer sequences (5' to 3') |
| --- | --- |
| *ACT*  *IAA32*  *SHI*  *VRN1*  *RAP2-7*  *RAV1*  *MYC2*  *C704B1*  *HACD2*  *CER1*  *KCS5*  *KCS10*  *TKPR2*  *KAN2*  *SPL5*  *SPL6*  *BT4* | Forward: ACCTTGCTGGTCGTGATCT  Reverse: GCAAGCTTCTCCTTCATGTC  Forward: GCAACAACTGCATCCACAGCTTAG  Reverse: CGACCAACAACTACACCGTCCATG  Forward: GGCGTTATGTCGGTTGGTGAGG  Reverse: GCAGCAAGTCCTACATCTCCTGTG  Forward: GCTTCCGCTAATGCCTTCAAATCG  Reverse: GGTCTTCAGATCGGTCTTCCTTGC  Forward: CGCACCACAATCTGGACCTTAGC  Reverse: GAGCAGGAGCAAACCTCTCAACC  Forward: TCGTGTCGGGTCGGGTTGTG  Reverse: ACCCCATCTCCCTCGTTCTCTTTC  Forward: GCCTGCGAATGGGAGAGAAGAAC  Reverse: ATTAGGCACCACAACACGAAGAGC  Forward: GACACCACTGCCACAACTCTGAC  Reverse: ACTCTCTTCTTTTGCCCGCTCTTC  Forward: GGAAACTATCCACGGAGCCATTGG  Reverse: TCCATGAGCAACTGCAACAACAAC  Forward: CATGGCGCATAGCTGGGATAGTG  Reverse: CATCGGTGGGAATTGTGAGTGGAC  Forward: CAGGAGGGCGTGCTGTAATTGAC  Reverse: GTATTGCCAAAGCGTTGCAGTGTC  Forward: TAACCCGACGCCATCTCTATCCG  Reverse: TCCCAGCACTACAGCCCATTCC  Forward: GGAGGATGGCAGAGGAAAATGGC  Reverse: CGTACTGGTTGGCTGAGGAGAAAG  Forward: AGCCAACACCACCTTCTCCTTTTC  Reverse: GGTGCTTCGCTTTGTCGGAAATC  Forward: TTGTCGTTGTTGCCGGAGTTGG  Reverse: TATGCCCAGCTAGTCGCCTACG  Forward: TGTCAGCAATGCAGCAGGTTCC  Reverse: TTGTGCCCAGCAAGACGTTTCC  Forward: ACTGTCTCACGCATATGTGGTTCC  Reverse: GCGGTGGCAAATGAGGGTCAG |
