# Supplementary material for: Full-length transcriptome analysis provides new insights into the early bolting occurrence in medicinal Angelica sinensis
Source: Sci Rep. 2021 Jun 21;11:13000. doi: 10.1038/s41598-021-92494-4 (PMC8217430; doi:10.1038/s41598-021-92494-4)
Supplement: Supplementary file 12 — Supplementary Table S6. [file 41598_2021_92494_MOESM12_ESM.docx]

**Supplementary Table S6** Gene comparison table from *Angelica sinensis* to *Arabidopsis thaliana*.

| **Transcript No in *A.sinensis*** | **Gene name in *A******rabidopsis*** |
| --- | --- |
| ZW_f2p60_1028_transcript35959_g2  ZW_f2p43_793_transcript38460_g1  ZW_f2p60_511_transcript41227_g1  ZW_f2p19_1272_transcript32782_g1  ZW_f2p60_453_transcript41663_g1  ZW_f2p57_992_transcript36342_g1  ZW_f2p60_2167_transcript17974_g1  ZW_f3p60_1303_transcript32309_g1  ZW_f2p60_1588_transcript28916_g4  ZW_f2p60_2162_transcript18097_g2  ZW_f2p60_919_transcript37041_g1  ZW_f3p60_746_transcript38893_g1  ZW_f3p60_551_transcript40897_g2  ZW_f4p60_1437_transcript30622_g1  ZW_f6p60_955_transcript36620_g1  ZW_f2p12_2445_transcript13673_g1  ZW_f2p31_1968_transcript21501_g1  ZW_f2p18_1549_transcript28950_g1  ZW_f3p60_1343_transcript31997_g2  ZW_f2p60_401_transcript41986_g1  ZW_f14p60_904_transcript37202_g1  ZW_f2p52_1442_transcript30588_g1  ZW_f3p60_1178_transcript33913_g2  ZW_f5p60_1782_transcript25303_g1  ZW_f6p60_1796_transcript24897_g1  ZW_f3p60_754_transcript38962_g3  ZW_f4p60_3648_transcript1971_g2  ZW_f6p60_1050_transcript35836_g1  ZW_f5p60_865_transcript37743_g1  ZW_f53p60_1712_transcript22917_g1  ZW_f6p60_1910_transcript22850_g2  ZW_f6p60_2366_transcript14524_g1  ZW_f2p26_2516_transcript11547_g1  ZW_f4p60_1564_transcript28777_g1  ZW_f3p60_2196_transcript17562_g1  ZW_f2p20_2706_transcript9238_g1  ZW_f2p60_1934_transcript22356_g1  ZW_f15p60_2375_transcript13796_g1  ZW_f2p13_1215_transcript33526_g1  ZW_f2p60_1872_transcript23372_g1  ZW_f8p60_1562_transcript27931_g1  ZW_f7p60_1331_transcript32135_g1  ZW_f2p60_1943_transcript22424_g1  ZW_f3p60_2257_transcript16290_g2  ZW_f4p60_3341_transcript3239_g1  ZW_f5p60_2702_transcript9955_g3  ZW_f3p60_2400_transcript13306_g2 | *IAA32*  *AUX22*  *SAU21*  *SHI*  *CYP707A2*  *MYB3*  *GH3.1*  *RAV1*  *RAP2-7*  *MYC2*  *ERF4c*  *ERF2*  *GASA11*  *VRN1*  *SPL5*  *SPL6*  *SPL8*  *SPL14*  *MYB35*  *QRT3*  *LAT52*  *TKPR1*  *TKPR2*  *CYP703A2*  *CYP704B1*  *WIN1*  *JMJ25*  *PAS2*  *HACD2*  *KCS5*  *KCS6*  *KCS10*  *CER1*  *CER26*  *HTH*  *KAN2*  *AB2E*  *AB25G*  *GDL34*  *GDL72*  *GDL79*  *GDL83*  *BT4*  *SR1IP1*  *At2g16250*  *At4g37250*  *At1g80870* |
